# Robotic-assisted gastrectomy for gastric cancer: single Western center results

**DOI:** 10.1007/s13304-020-00896-2

**Published:** 2020-10-14

**Authors:** Luigi Marano, Alessia D’Ignazio, Luca Resca, Daniele Marrelli, Franco Roviello

**Affiliations:** Department of Medicine, Surgery and Neurosciences, Unit of General Surgery and Surgical Oncology, University of Siena, Strada Delle Scotte, 4, 53100 Siena, Italy

**Keywords:** Gastric cancer, Robotic gastrectomy, Survival, D2 lymphadenectomy

## Abstract

A robotic approach to abdominal surgery procedures may improve postoperative outcomes compared to either open or laparoscopic approaches. The role of robotics for gastric surgery, however, is still being evaluated. A retrospective review of the prospectively maintained database for robotic gastric surgery at University of Siena between 2011 and 2020 was conducted. Data regarding surgical procedures, early postoperative outcomes, and long-term follow-up were analyzed. 38 patients underwent robotic partial or total gastrectomy. Conversion to open occurred in two patients (5.2%) due to locally advanced disease as well as difficult identification of primary lesion. Postoperative morbidity was 13.1% while no postoperative mortality was registered. The mean length of operation was 358.6 (220–650) minutes and the mean number of retrieved lymph nodes was 35.8 (range: 5–73). The median OS of all population was 70.9 months. The median 5-year OS for the patients with positive nodes was worse than that of patients without metastatic lymph nodes [51.4 months (95% CI 35.5–67.4) vs. 79.5 months (95% CI 67.1–91.8); *p* = 0.079]. The interesting results including postoperative morbidity as well as mortality rate, the surgical outcomes, and the 5-year OS, were to be acceptable considering the data recorded by previous studies on robotic gastrectomy. This study demonstrated that robotic gastrectomy is feasible and can be safely performed. However, further follow-up and randomized clinical trials are required to confirm the role of a robotic approach in gastric cancer surgery.

## Background

During the last decades, several studies have provided evidence that laparoscopic surgery for gastric cancer is technically safe and that it leads to better short-term outcomes than conventional open gastrectomy for early-stage gastric cancer. However, a safer D2 spleen-preserving laparoscopic gastrectomy for the treatment of advanced gastric cancer did not meet the same success and is currently available only in high-volume centers. Technical difficulties due to total gastrectomy procedure as well as D2 lymphadenectomy, entailing the removal of node stations along the celiac trunk, left gastric artery and hepatic pedicle, are advocated as limiting factor of laparoscopic surgery diffusion. To overcome some intrinsic limitations of the traditional laparoscopic approach, robotic approach is advocated by some authors as able to facilitate complex reconstruction after gastrectomy and the lymph node dissection, so as to assure oncologic safety also in advanced gastric cancer patients. The first robot-assisted gastrectomy (RAG) was reported from Hashizume et al. [1] and Giulianotti et al. [2] in the 2003. Since then, several authors worldwide reported their experience on RAG for cancer and the largest single institution series investigating clinical and oncological outcomes include: Song et al. [3] in 2009, Jiang et al. [4] in 2012, Liu et al. [5] and Park et al. [6] in 2013, Tokunaga et al. [7] in 2015. Recent evidence supports the feasibility and the efficacy of robotic partial and total gastrectomy, showing advantages in terms of decreased blood loss and the higher number of retrieved lymph nodes [8]. Despite the perioperative outcomes well assessed by previous researchers, few reports are focused on the long-term outcomes of robotic gastrectomy. For these reasons the oncological efficacy of robotic approach remains controversial. The aim of this study is to describe our experience, surgical techniques and the short- as well as long-term outcomes of a consecutive series of full robotic gastrectomies using the Da Vinci Surgical System.

## Patients and methods

### Patients

Between January 2011 and April 2020, 38 patients with pathological confirmed gastric cancer underwent curative resection using robotic surgery (Da Vinci Si and, since 2015, Da Vinci Xi; Intuitive Surgical, Inc., Sunnyvale, CA, USA) at University of Siena, Surgical Oncology Department. Single surgeon, expert in digestive surgery with an extensive advanced laparoscopic experience and completed robotic surgery training program, performed all of the procedures with a D2 gastrectomy following the Japanese Classification of Gastric Cancer (JCGC) [9] as well as the Italian Research Group for Gastric Cancer (GIRCG) guidelines for diagnosis and treatment of gastric cancer [10]. The preoperative protocol included esophagogastroduodenoscopy with core-needle biopsy and thoraco-abdominal contrast-enhanced CT scan. The prospectively maintained database included details about patients, pathologic reports according to the 8th TNM edition [11], details of procedure, postoperative complications, and follow-up outcomes. Post-operative mortality was defined as death within 90 days of surgery, while postoperative morbidity was recorded and scored according to the Clavien-Dindo classification system [12]. Patients were regularly followed up after the surgery either in the surgical or oncological department with blood tests (including tumor markers) and computed tomography every 6 months the first 2 years, every 12 months from year 3 and 5, and yearly after that date, or on demand at any time according to clinical status.

All patients gave the informed consent for data recording in the registry and were treated according to a multidisciplinary recommendation. Due to the retrospective nature of the analysis of the anonymized data, no institutional review board approval was needed.

### Surgical techniques

The patient is placed in the supine position on a split table. A nasogastric tube is inserted for gastric decompression. Pneumoperitoneum induction is carried out through the left upper quadrant using a Veress needle with an intra-abdominal pressure of 13 mmHg. An 8-mm robotic port is placed just lateral to the left side of umbilicus for 30° optics. Under direct vision, three 8-mm robotic trocars are inserted: two in the upper abdomen at the midclavicular line on the left and on the right, and one at the right anterior axillary line for liver retraction. After placement of the ports, the patient is positioned in a reverse Trendelenburg’s position at approximately 15–20°. The Da Vinci Surgical System is moved to the operative table above the patient’s head and the operative arms are connected to the ports. During the procedure, a 12-mm port is used by the assistant surgeon between the left robotic port and the camera port for the introduction of aspiration, clip applier, sutures and stapler.

### Robot-assisted sub-total gastrectomy (RASG)

**Fig. 1:**
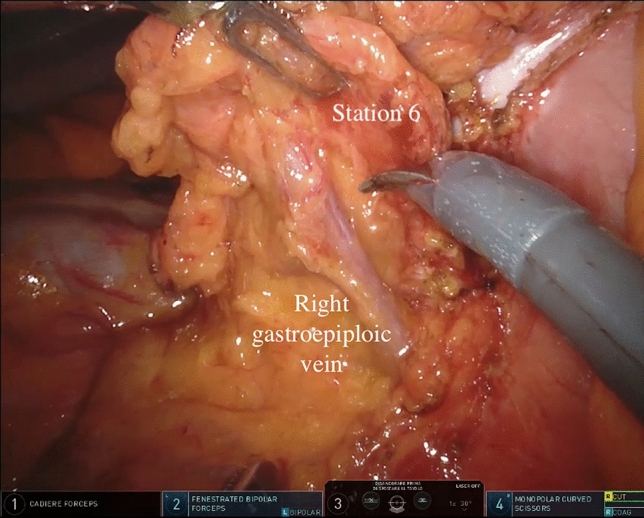
Lymph node dissection of station 6

**Fig. 2:**
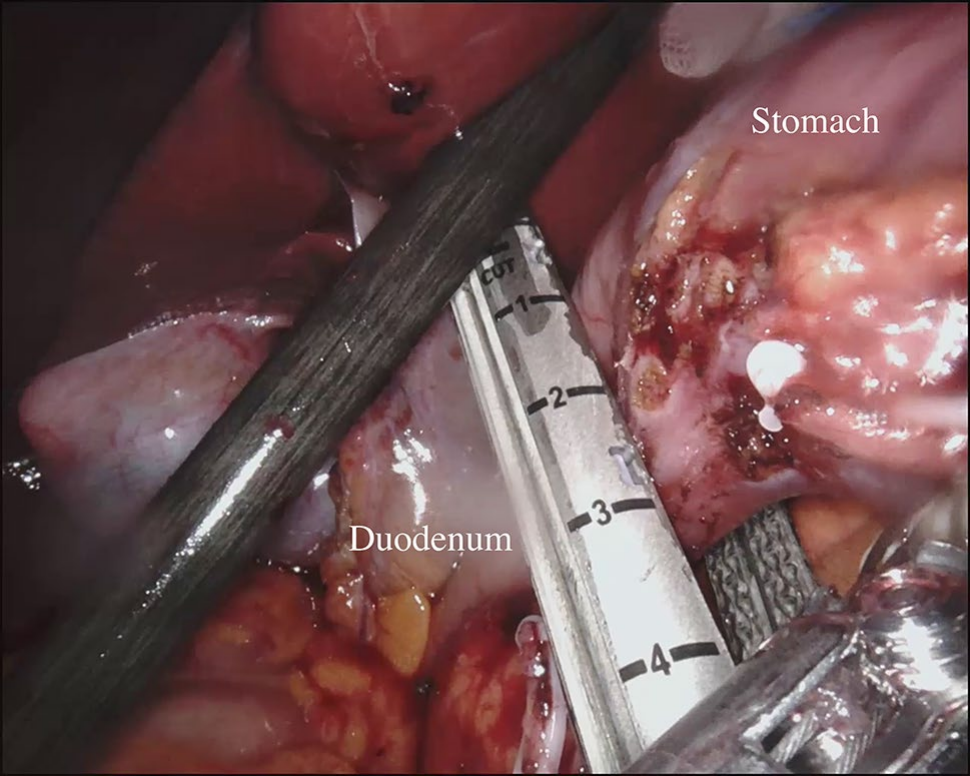
Duodenal transection

**Fig. 3:**
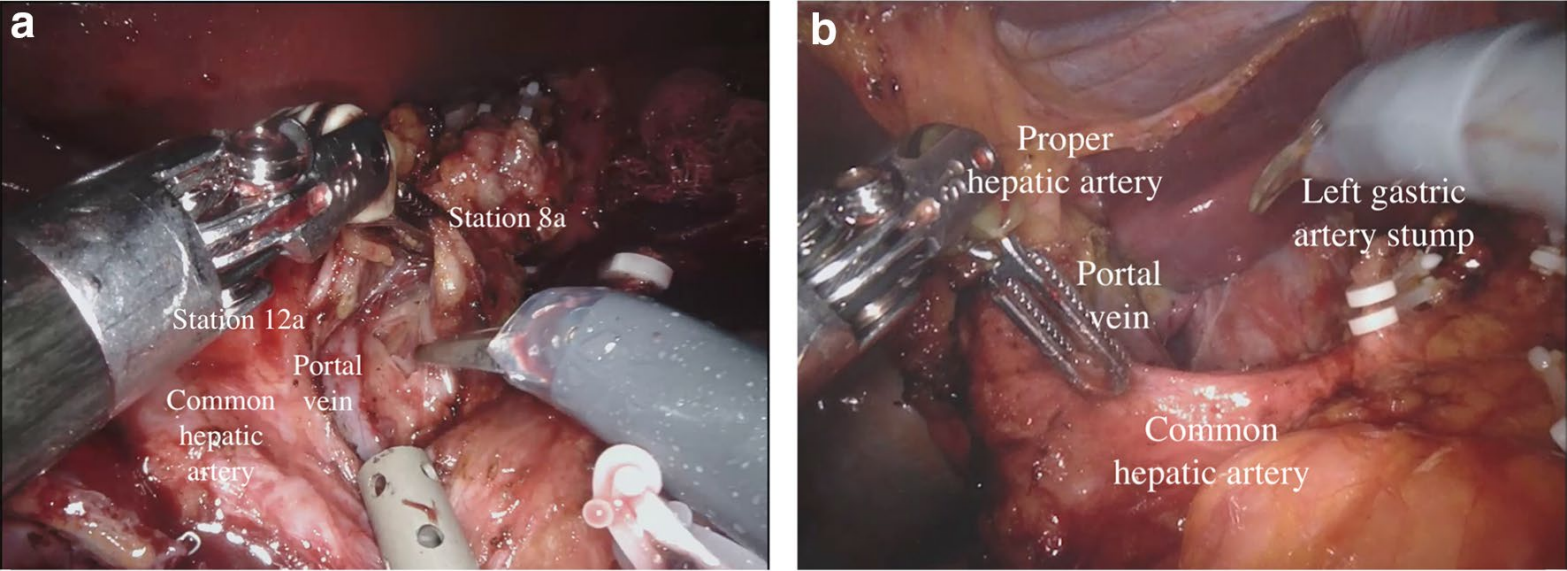
Lymph node dissection of stations 8a and 12a. **a** During lymphadenectomy; **b** post-procedure celiac region

**Fig. 4:**
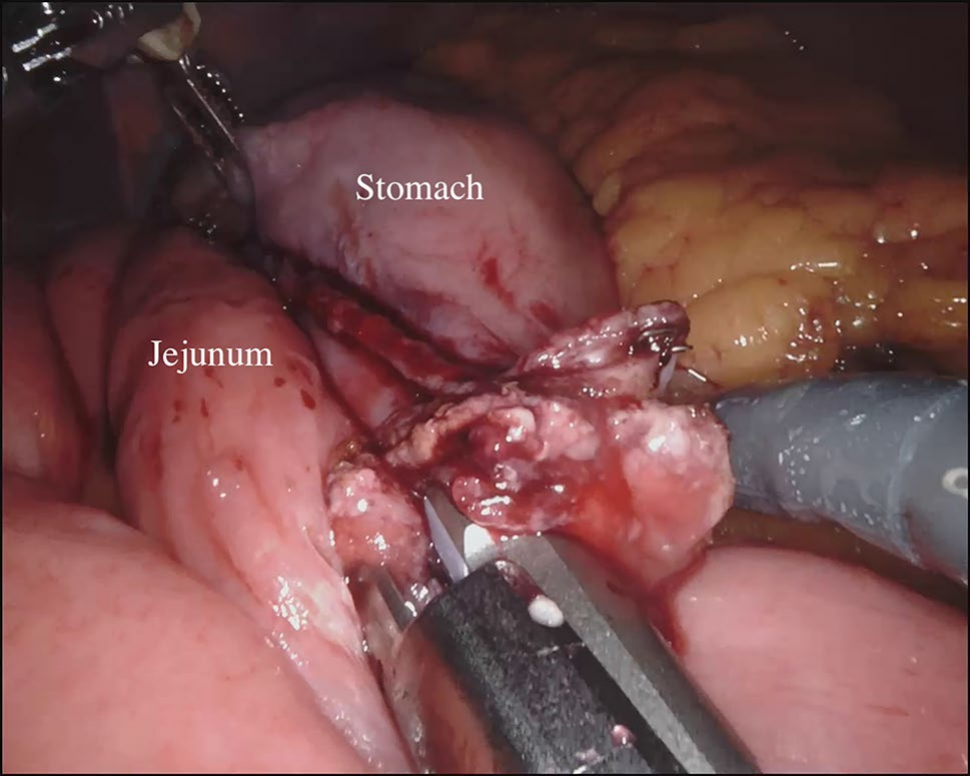
Billroth II anastomosis

During the robot-assisted subtotal gastrectomy, the colon-epiploic detachment is first obtained and then dissected in the direction of the lower pole of the spleen and distally toward the pylorus using monopolar scissors and bipolar forceps. The dissection is then continued to the more distal short gastric vessels, which are sectioned at their roots with bipolar coagulation and clips including lymph nodes stations 4sb and 4d. The right gastroepiploic vessels are then dissected en bloc with lymphatic tissue (Station 6) (Fig. 1). The lesser omentum is then opened from pars flaccida to the hepatic pedicle. With this dissection, we remove the lymph nodes near the lesser gastric curve (station 3). Next, the proper hepatic artery is cleaned to identify the right gastric artery. This maneuver allows to dissect the lymph nodes of station 5. Then, the release of the first part of the duodenum is completed and its transection can be performed using a powered linear stapler (Signia™ stapling system, Medtronic, Minneapolis, MN, USA) (Fig. 2). Lymphadenectomy of major vessels is carried out starting from the hepatic hilum toward the celiac trunk, removing the station 12a and 8a (Fig. 3). Once the celiac trunk is released, the left gastric artery can be easily identified and ligated, and the lymph nodes of station 7 dissected. The left gastric vein is also ligated. The lymphadenectomy of the splenic artery can be performed according to the tumor characteristic, retrieving the stations n.10 and n.11p. Once the lymphadenectomy is completed, the stomach is transected at its proximal third by the assistant surgeon using a powered linear stapler. The specimen (stomach, omentum and lymphatic tissue) is placed into a large endobag and retrieved through a median mini-incision. The digestive tract is restored by an intracorporeal antecolic Billroth II gastrojejunal anastomosis (Fig. 4).

### Robot-assisted total gastrectomy (RATG)

The surgical steps are the same as for distal gastrectomy, except for the following: division of the short gastric vessels and dissection along the gastro-splenic ligament, lymphadenectomy, digestive tract reconstruction.

The gastrosplenic ligament is separated up to the left side of the esophageal hiatus by dividing the short gastric vessels from the surface of the spleen, to dissect the nodes station 4sa and 2 and to mobilize the upper part of the greater curvature of the stomach. To achieve a correct D2 lymphadenectomy, the dissection involves the nodes on the distal splenic artery (station 11d) and the splenic hilum (station 10); the spleen is preserved, unless there is massive lymphatic metastasis of the hilum or direct infiltration of the organ. The abdominal esophagus is then prepared dissecting the diaphragmatic crura to achieve a safe resection margin, from an oncologic point of view, and a good stump for the anastomosis. If necessary, the hiatus is widened with an anterior incision using the robotic monopolar scissors and the dissection is conducted around the distal esophagus to retrieve the lower posterior mediastinal nodes (station 110).

The esophagus is divided from the assistant surgeon using a powered linear stapler. The esophagojejunal anastomosis can be performed both with a circular stapler or robot-assisted hand-sewing technique. In the first case, a purse string is fashioned on the esophageal stump by robotic handsewn technique; the anvil of circular stapler is inserted into the esophageal stump, and the purse string is closed. The circular stapler is then inserted into the jejunal loop and introduced inside the abdomen through the left pararectal mini-laparotomy. When the stapler is inside the abdominal cavity and the pneumoperitoneum is re-established, the end-to-side esophagojejunostomy is performed under standard laparoscopic control. The shrimp of jejunal loop is closed by a powered linear stapler.

### Statistical analysis

Descriptive statistics were reported as median with minimum and maximum values or frequency with percentage. Overall survival and recurrence free survival analyses were performed with the use of the Kaplan–Meier estimation method and compared using the log-rank test. A *p* value < 0.05 was considered statistically significant. All statistical analyses were performed using the SPSS version 26.0 software package (IBM Corp., Chicago, IL, USA).

## Results

### Patient characteristics

The study group included a totality of 38 patients, 14 females and 24 males, with a mean age of 68.8 years (range 43–87 years). Cancer lesions were localized in the lower third of the stomach in 52.6% of cases, in the middle third in 28.9%, and in the upper third in 18.4%. Of these, the 10.5% were cardias tumor, two cases Siewert II, two cases Siewert III.

The patients underwent distal subtotal gastrectomy were 31 (81.6%), total gastrectomy 7 (13.5%). In four patients with type II and III Siewert lesions, we preferred a robotic transhiatal approach. There were no patients classified as ASA I, 17 patients ASAII (44.7%), 21 patients ASA III (55.3%). Pathological reports reveal that, according to the pTNM 8th edition, 18 (50%) patients were classified as stage I disease, 9 (25%) stage II, 8 (22.2%) stage III, and 1 case (2.8%) stage IV. The clinicopathology characteristics were summarized in Table 1.

**Table 1:** Patient characteristics

	Overall *n* = 38 (%)
Gender	
Female	14 (36.8%)
Male	24 (62.2%)
Age in years, median (range)	68.6 (43–87)
BMI, median (range)	27 (20–37)
ASA score	
1	–
2	17 (44.7%)
3	21 (55.3%)
4	–
Location of tumor	
Upper third	7 (18.4%)
Middle third	11 (28.9%)
Lower third	20 (52.6%)
Tumor stage	
I	20 (52.6%)
II	8 (21.1%)
II	9 (23.7%)
IV	1 (2.6%)
Histological Lauren subtype	
Intestinal	20 (52.6%)
Diffused and mixed	18 (47.4%)

*BMI* body mass index; *ASA* American Society of Anesthesiologists

### Perioperative outcomes

Surgical results indicated that 35 (94.8%) patients had R0 resections while no R2 resections were registered. Only two R1 resections (5.2%) were observed on the basis of final pathology examination, due to infiltrated distal margins. Conversion to open surgery was needed in two patients (5.2%). In the first case, the conversion was due to the difficult identification of the exact location of the tumor (clinical T1), despite the previous endoscopic marking; the second conversion case was due to invasiveness of the tumor: the involvement of the left diaphragmatic pillar, bulky nodes at celiac trunk as well as at hepato-duodenal ligament. No postoperative mortality was registered, while complications occurred in five patients (13.1%). According to Clavien-Dindo classification, three patients had grade II complications and two patients had grade IIIb complications. Particularly, one patient had a pancreatitis and a massive involvement of the anastomosis into the inflammatory process with subsequent stenosis. Additionally, the other one had a pancreatitis with abdominal abscess requiring a surgical reintervention. In the others three patients, leaks of esophago-jejunal anastomoses were observed: in all cases the resolution occurred with conservative therapy as parenteral nutrition and nihil per os (Table 2).

**Table 2:** Perioperative and oncological outcomes

	Overall *n* = 38 (%)
Surgical procedure	
Total gastrectomy	7 (18.4%)
Subtotal gastrectomy	31 (81.6%)
Mean time of surgery (range)	358.6 (220–650)
Type of resection	
R0	36 (94.8%)
R1	2 (5.2%)
R2	0
Mean hospital stay in days, (range)	9.4 (5–22)
Complications	
Anastomotic leak	3 (7.9%)
Others surgical complications	2 (5.3%)
Non-surgical complications	–
Mean of total retrieved nodes (range)	35.8 (5–73)
Recurrence	
Liver	1 (2.6%)
Peritoneum	4 (10.5%)
Others	1 (2.6%)
None	32 (84.2%)

### Oncological outcomes

The mean number of retrieved lymph nodes was 35.8 (range: 5–73). The harvested lymph nodes were 28.7 (range 15–41) in D1 dissection and only one lymph nodes results positive for cancer; 31.9 nodes ( range 10–63) in D2 dissection and a mean of 2.7 lymph nodes results positive (range 0–13); 39.8 ( range 5–73) in D3 and 4.7 were positive (range 0–41). The recurrence occurred in six patients (15.7%): one (2.6%) patients had liver recurrence, 4 (10.5%) peritoneum, one (2.6%) patients had an adrenal recurrence (Table 2).

### Survival analysis

**Fig. 5:**
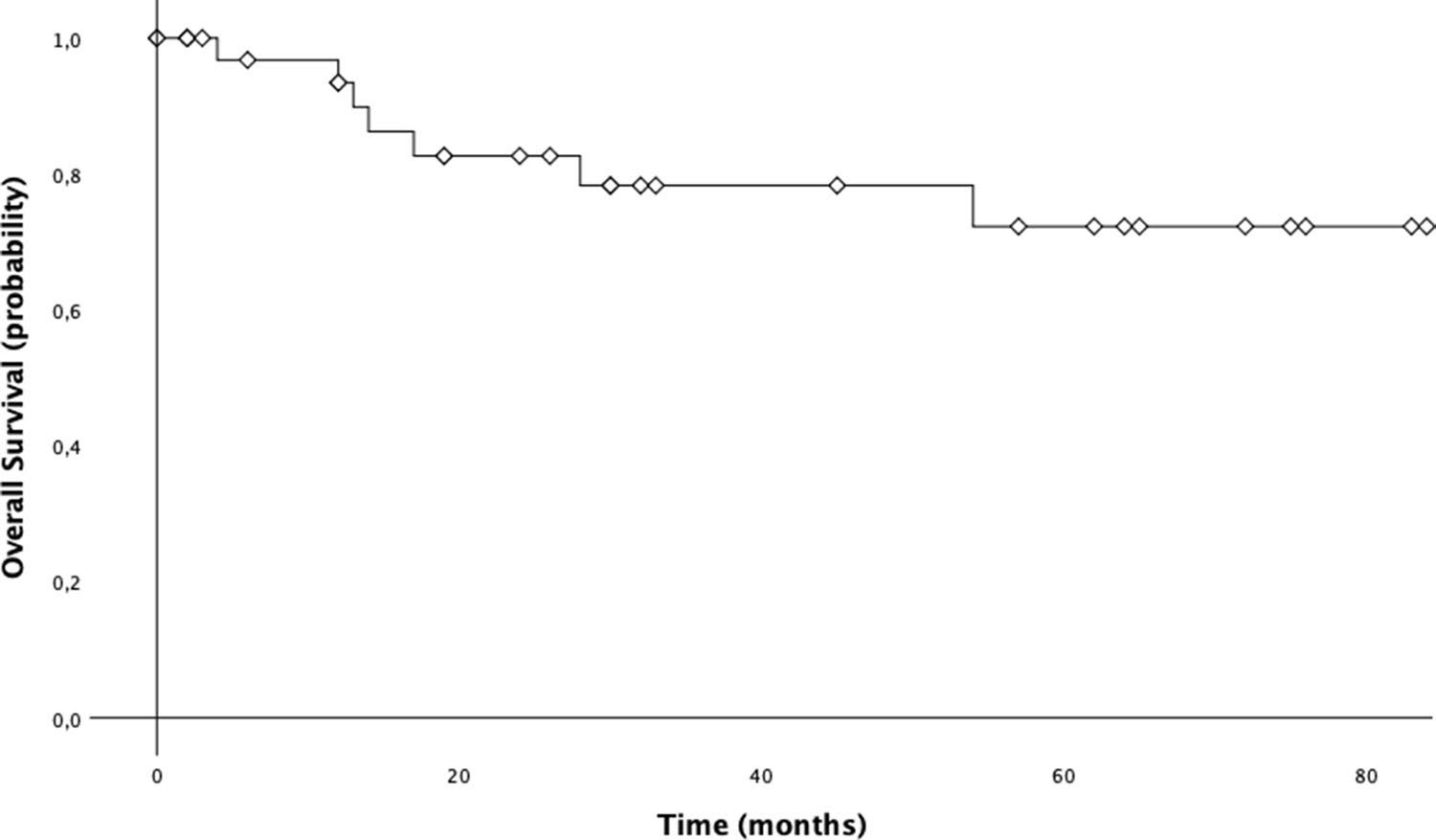
5-year overall survival (OS) of patients with gastric cancer treated with robotic gastrectomy

**Fig. 6:**
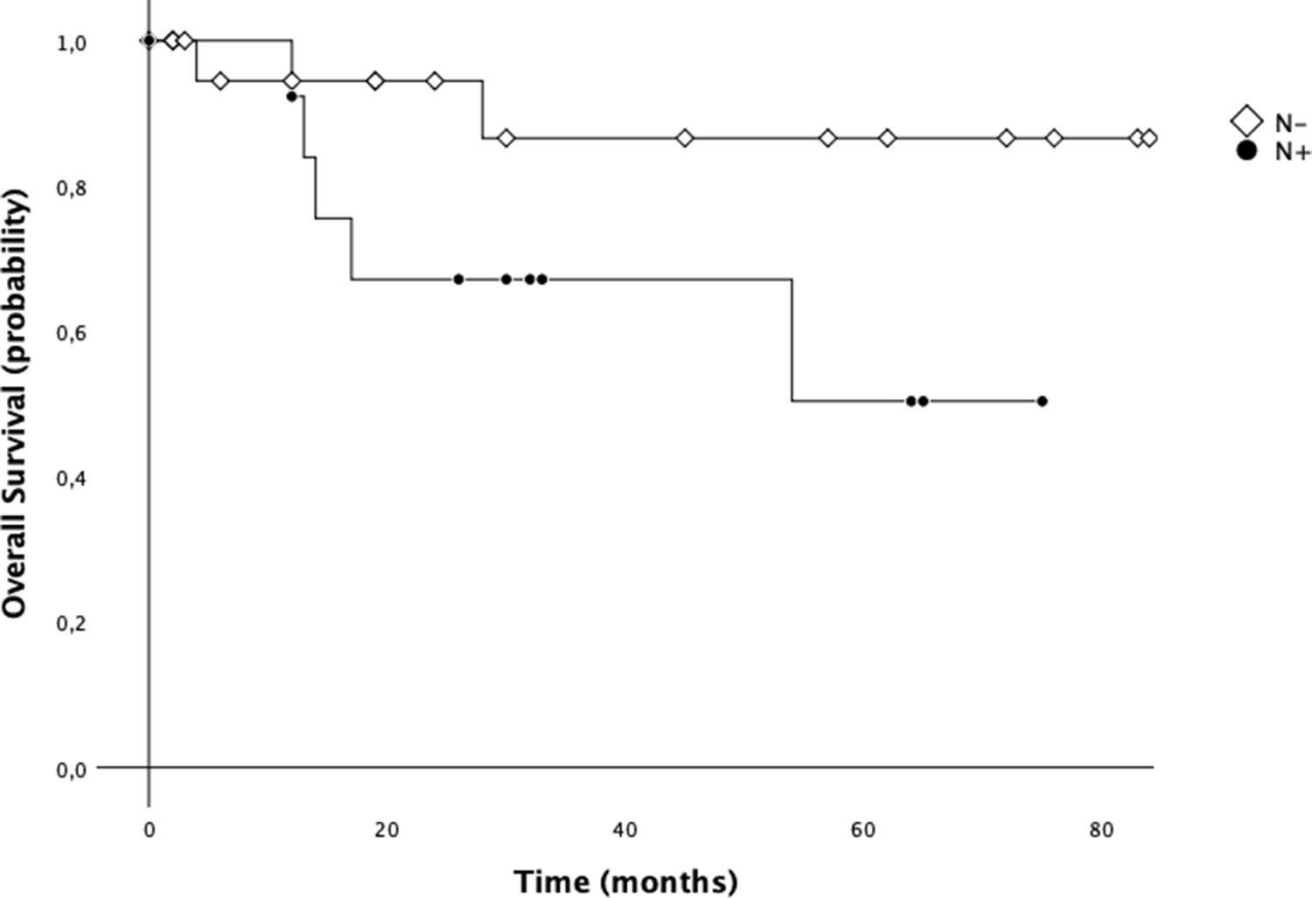
5-year overall survival (OS) of patients with gastric cancer treated with robotic gastrectomy according to N status

**Fig. 7:**
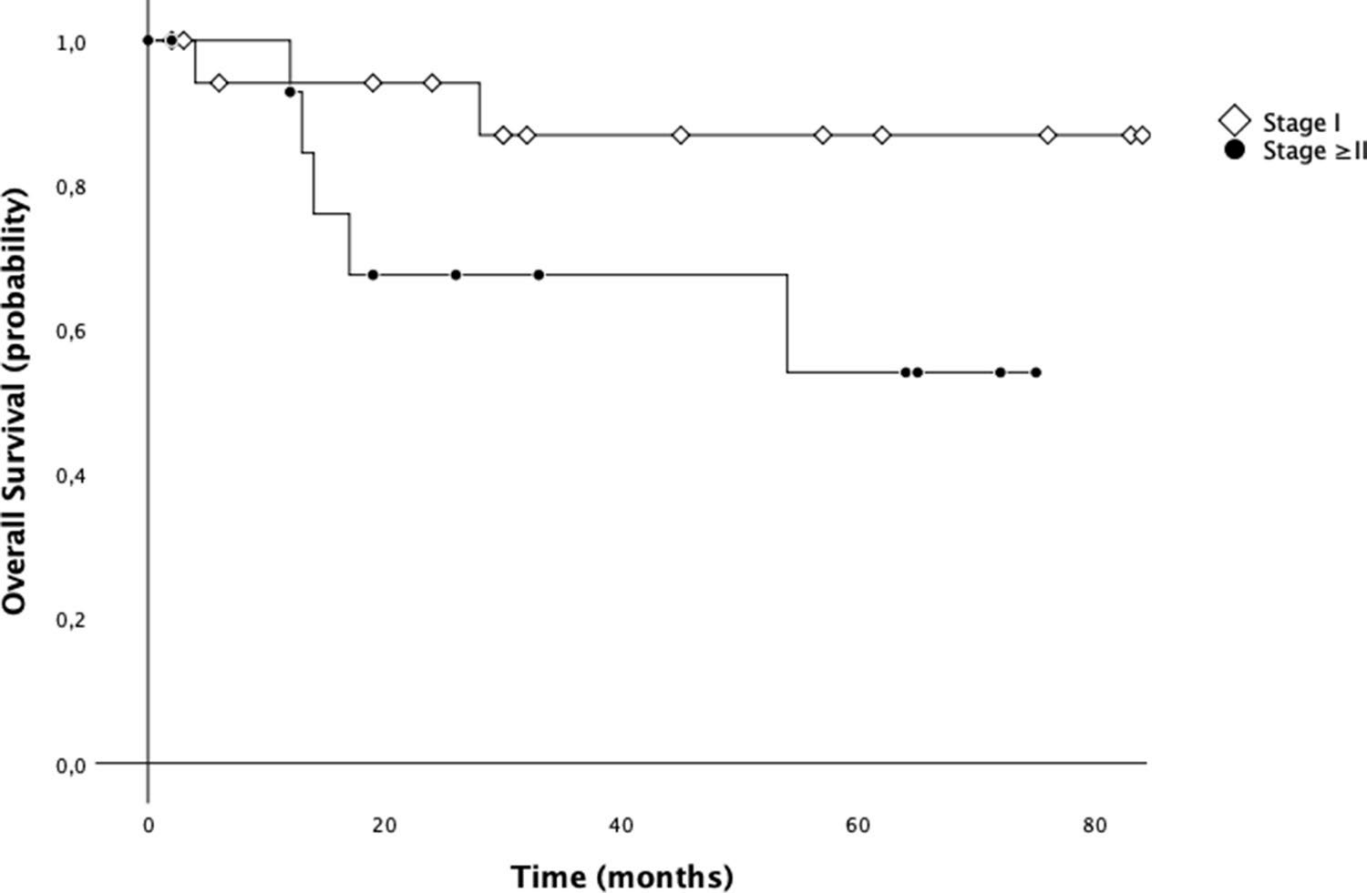
5-year overall survival (OS) of patients with gastric cancer treated with robotic gastrectomy according to stage

Data of 29 patients from January 2011 to December 2017 were considered for survival analysis. Cumulative 3-year OS was 78.3% and 5-year OS was 72.3%. The median OS of all population was 70.9 months (CI 95%: 59.2–82.6) (Fig. 5). We also investigated the differences in OS between patients with and without lymph node metastasis. On this address, median 5-year OS for the patients with positive nodes was worse than that of patients without metastatic lymph nodes [51.4 months (95% CI 35.5–67.4) vs. 79.5 months (95% CI 67.1–91.8); *p* = 0.079] (Fig. 6). Additionally, median 5-year OS for the patients with stage ≥ II was worse than that of patients with stage I [52.4 months (95% CI 36.6–68.1) vs. 79.6 months (95% CI 67.3–91.8); *p* = 0.093] (Fig. 7).

## Discussion

Our Western single center activity shows encouraging results, since on a total of 38 enrolled patients treated with robotic approach, no postoperative mortality was registered while complications occurred in 13.2% of patients. Interestingly, median overall survival (OS) was 70.9 months, with a 5-year OS rate of 72.3%.

Despite several reports regarding the safety and feasibility of robotic gastrectomy, only few comparative analysis investigated RAG vs. laparoscopic and/or open gastrectomy. Several studies comparing robotic with open and laparoscopic approach are retrospective with limited sample size. Only multicentric comparative study by Kim et al. [12] was prospectively conducted: they compared a total of 434 gastric cancer patients submitted to robotic and laparoscopic gastrectomy (223 vs. 211, respectively), showing similar overall complications rate with no operative mortality in either group. On the other hand, operative time and management cost resulted significantly higher in robotic group, despite the duration of the hospital stay is significantly reduced [13]. In some cases, potential clinical advantaged were highlighted. Kim et al. [12] and Suda et al. [14] showed a statistically significant improvement of the postoperative morbidity rate in gastric cancer patients submitted to RAG compared to LAG. Particularly, Suda et al. stated that local (particularly pancreatic fistula, robotic 0% vs. conventional laparoscopy 4.3%, *P* = 0.029) rather than systemic complication were reduced using the robotic platform [12]. Seo et al. reported advantages of RAG in comparison to LAG in terms of reduction of postoperative pancreatitis or pancreatic fistula [15]. The more gentle and steady pancreatic compression by means of robotic instruments during the suprapancreatic lymph nodes dissection could explain these results. Additionally, Seehofer et al. [16] demonstrated that heat production in bipolar devices was lower than ultrasonic straight cutting devices. On this basis, the major advantages of robotic bipolar forceps with endowrist technologies were the containment of lateral thermal damage and potential injury to adjacent organs. This leads to a safer tissue division during the dissection close to susceptible organs like pancreas. Our data seem to be in line with these results, since on a total of 38 patients we registered only 2 cases (5.2%) of pancreatic fistula requiring surgical intervention for subsequent abdominal abscess.

The morbidity rate by our series reached the 13% with no postoperative mortality registered, in line with the morbidity rate between 4.9 and 13% and a mortality rate of 0–6% reported in literature [14, 17, 18]. Junfeng et al. [16] retrospectively compared 120 vs. 394 gastric cancer patients who had undergone RAG and laparoscopic assisted gastrectomy (LAG), respectively, revealing similar results. Additionally, the authors showed that the numbers of harvested lymph nodes were notably superior in the RAG group. Similarly, Kim et al. [12] commented that robotic approach seemed to be advantageous over laparoscopy in performing the nodes dissection at the second level, mainly as regard the suprapancreatic and splenic artery nodes. This evidence seems to support the advantage of robotic surgery over LAG in its ability to perform a more complete D2 lymphadenectomy, overcoming one of the greatest surgical drawbacks of the laparoscopy in the curative treatment of gastric cancer. In accordance with the results of the present study, also the reported data from literature confirmed the safety and feasibility of RAG for gastric cancer, reporting an adequate number of retrieved lymph nodes [17]. Coratti et al. [18] were the first to report long-term survival data specifically referring to gastric cancer patients treated with robotic approach. They analyzed survival results in a group of 98 patients with either early as well as advanced gastric cancer submitted to RAG, as in the present study. As we found, they registered a cumulative 5-year survival rate of 73.3%. Son et al. [19], in a median long-term follow-up of 70 months, did not find significant differences in overall survival and disease-free survival between the robotic and laparoscopic groups. Specifically, the authors reported a 5-year overall survival rate of 89.5% for the robotic group, which was not statistically significant different with respect to the rate revealed in the laparoscopic group (91.1%). Results from our experience highlighted a 5-year OS of 72.3%, comparable with outcomes from other Authors. Another interesting issue regards the demonstration of robotic gastrectomy validity in carrying out an adequate extended lymphadenectomy leading to potential oncological benefit. In our study, we obtained a mean of retrieved lymph nodes equal to 35.8 (5–73). Similarly, in a recent paper, Jiang Y et al. [20] reported the mean number of retrieved lymph nodes equal to 33, along with the results of the previous robotic, laparoscopic and open gastrectomy [21] indicating an adequate lymph node dissection of radical lymphadenectomy. Furthermore, we also investigated the differences in OS between patients with and without lymph node metastasis. On this address, median 5-year OS for the patients with positive nodes was worse than that of patients without metastatic lymph nodes (51.4 months vs. 79.5 months) even if statistical significance was not reached.

Anyway, these results, albeit initial, are promising, but the robotic approach has not yet been solidly proved and its validation is still a long way for all gastric cancer patients. Many prospective clinical trials are ongoing, predominantly from Eastern Countries, focused on the early stages cancers as MONA LISA study (JCOG 1907). Unfortunately, due to inadequate long-term follow-up results and a limited number of studies to date available, larger and randomized prospective trials are required to draw definitive conclusion in Western Countries [20]. On this route, the interest of Western referral centers for Gastric cancer is increasingly oriented to a prospective collection of data, as in the context of UGIRA (The Upper GI International Robotic Association) for esophageal cancer surgery [22]. The major limitations of our single center study are the retrospective nature of the data and the little number of enrolled cases. Nonetheless, this is one of the most important reported experience from Italian high-volume center with specific competence in gastric malignancies, published so far.

## Conclusions

In conclusion, the interesting results including postoperative morbidity as well as mortality rate, the surgical outcomes, and the 5-year OS, were to be acceptable considering the data recorded by previous studies on robotic gastrectomy. This study demonstrated that robotic gastrectomy is feasible and can be safely performed. However, further follow-up and randomized clinical trials are required to confirm the role of a robotic approach in gastric cancer surgery.

## Data Availability

Not applicable.
